# The Association Between Medicaid Expansion and Diabetic Ketoacidosis Hospitalizations

**DOI:** 10.7759/cureus.30631

**Published:** 2022-10-24

**Authors:** Zehra Tekin, Meryem Saygili

**Affiliations:** 1 Endocrinology, Diabetes and Metabolism, Christus Trinity Clinic, Tyler, USA; 2 Social Sciences/Economics, The University of Texas at Tyler, Tyler, USA

**Keywords:** difference-in-differences, health economics, medicaid expansion, diabetic ketoacidosis hospitalizations, diabetes

## Abstract

Background and objective

Diabetic ketoacidosis (DKA) is a potentially fatal complication of uncontrolled diabetes and remains a significant source of morbidity and mortality even though it is considered preventable. Diabetes is a chronic illness that requires constant monitoring and regular check-ups. Delaying or foregoing necessary diabetes care due to a lack of health insurance can result in severe complications. The Affordable Care Act (ACA) Medicaid expansion is intended to increase access to healthcare and improve health outcomes. This study aimed to examine the relationship between the ACA Medicaid expansion and hospitalizations with DKA.

Methods

This retrospective cross-sectional study used discharge records from 2010 to 2017 for hospitals in Texarkana, located on the border of Texas and Arkansas. The study employed a difference-in-differences method. Patients from Arkansas, which expanded Medicaid in 2014, constituted the treatment group, while those from Texas, which did not adopt the expansion, were the control group. A triple difference methodology was used to compare the impact of the expansion on patients with different socioeconomic backgrounds. The main outcome measure was DKA per 1000 discharges.

Results

A total of 89,184 inpatient discharges from Texarkana hospitals were analyzed; 43,286 patients were from Arkansas (48.54%) and 45,898 (51.46%) were from Texas. Even though DKA cases increased from pre-expansion (2010-2013) to post-expansion (2014-2017) period among patients from Arkansas (by a mean of 4.33) and Texas (by a mean of 8.28), the increase was milder among Arkansas patients with an adjusted decrease of 4.17 per 1000 discharges (95% CI: -5.04 to -3.31; p<0.001), implying a 42% lower risk of hospitalizations with DKA compared to the baseline averages. The triple difference analysis suggested that the decrease in incidences was more pronounced for patients from low-income areas with an adjusted decrease of 13.47 per 1000 discharges (95% CI: -22.45 to -4.49; p=0.003).

Conclusions

Based on our findings, Medicaid expansion decreases hospitalizations with DKA, presumably due to better monitoring and care of diabetes made possible by increasing access to healthcare among individuals with low incomes.

## Introduction

Diabetes is one of the major health problems in the US, with 10.5% of the nation's population suffering from it [1], and is predicted to remain so in the future [2]. Diabetic ketoacidosis (DKA) is a potentially fatal complication of uncontrolled diabetes and continues to be a significant source of morbidity and mortality even though it is considered preventable [3]. Constant monitoring and regular check-ups are recommended for people with diabetes [4]. Patients who receive preventive procedures and sufficient education are less likely to visit the emergency room [5]. Delaying or foregoing necessary diabetes care due to a lack of health insurance coverage can result in severe complications [6].

The state-level Medicaid expansions play a central role in the Affordable Care Act (ACA) and are intended to increase access to healthcare and improve health outcomes [7-10]. The expansion has made all individuals with income up to 138% of the federal poverty line eligible for Medicare. However, the June 2012 US Supreme Court ruling effectively made Medicaid expansion an option for states.

Texas is one of the few states that has not expanded Medicaid as of 2022, while all its adjacent states, including Arkansas, have done so. Arkansas expanded Medicaid through a Section 1115 waiver. This approach, known as the “private option”, uses Medicaid funds to purchase private health plans on the state’s marketplace. A two-state city, Texarkana, represents an unusual example where people living in the Arkansas half of town gained access to the benefits of the Medicaid expansion, but nothing changed for those on the Texas side [11]. The current study sought to examine the association between Medicaid expansion and hospitalizations with DKA. While the literature has some studies exploring the impact of Medicaid expansion on diabetes and its management [12-19], none of them have looked specifically into DKA hospitalizations.

## Materials and methods

Data collection

Quarterly inpatient hospital discharge records from Texarkana for the period from 2010 to 2017 were obtained. The data were sourced from the Texas Department of State Health Services (DSHS) Hospital Discharge Database [20]. The hospitals located on the Texas side serve patients from both the Texas and Arkansas side of the city. The data captured hospital discharges accurately since all hospitals (except for a behavioral health hospital) are on the Texas side. There were around 90,000 inpatient records over the eight-year study period, with an average of 11,000 discharges per year. This study was exempt from Institutional Review Board approval because data are anonymized and publicly available.

Variables

The outcome variable was DKA hospitalizations per 1000 discharges. We identified DKA hospitalizations based on the principal and secondary diagnosis codes defined by the International Classification of Diseases (ICD). The dataset reports ICD-9 codes until the third quarter of 2015 and ICD-10 codes afterward. The DKA indicator variable takes the value of one, if the principal or secondary diagnoses have the ICD-9 codes of 250.10, 250.11, 250.12, 250.13, or the ICD-10 codes of E10.1, E10.10, E10.11, E11.1, E11.10, E11.11, E13.1, E13.10, E13.11, E08.1, E08.10, E08.11, E09.1, E09.10, E09.11.

Several patient characteristics, including age, sex assigned at birth, race, and expected payment sources were available; 27 age categories were reported in the original data. We excluded patients older than 65 years because the Medicaid expansion is unlikely to affect them as they are already eligible for Medicare. We also excluded patients younger than five years since we do not have any DKA incidence among those, and many of those discharges were newborn records. We were left with 16 age categories, and of those, three were for patients with HIV and drug/alcohol use. Those patients are categorized separately into broader age ranges due to confidentially concerns. The race categories are Black, White, and other. We classified discharges based on the expected primary source of payment into four categories. The codes for Medicare Part A and B are included in Medicare; the code for Medicaid is recorded in Medicaid; self-pay, charity, indigent, or unknown make up the Uninsured; and private insurance is included in the Private category. We calculated the Charlson Comorbidity Index for each discharge and obtained the median household income of patients’ zip code of residence from the American Community Survey.

Statistical analysis

We first reported the sample statistics (distributions of categorical variables and averages of numerical variables) for the treatment and the control groups in the pre-treatment (2010-2013) and the post-treatment (2014-2017) periods. The patients from Arkansas were the treatment group and those from Texas were the control group.

We used a multivariate difference-in-differences model to assess whether DKA rates changed differentially for the treatment and control groups from the pre-treatment to the post-treatment period. The details on all the empirical equations estimated and a formal test of the parallel trends assumption, which is crucial for the validity of difference-in-differences analyses, are provided in the Appendix.

The ACA Medicaid expansion is intended to address high uninsured rates among adults who are ineligible for Medicare but have no access to health insurance through employment or have limited income to purchase health insurance on their own. We expect that the Medicaid expansion would have a bigger impact on individuals with low income. Thus, we grouped patients based on the median income of the zip code of their residence. We created an indicator variable capturing patients from zip codes where median incomes are lower than the 10th percentile of the median income distribution in the data ($20,864). We estimated a difference-in-difference-in-differences model to examine if the individuals from economically disadvantaged areas in the Arkansas side of the city had a different outcome after the Medicaid expansion compared to those on the Texas side.

Several sensitivity analyses were conducted. The data included patients younger than 18 years of age. Even though Medicaid expansion does not have a direct impact on those children since most of them are already eligible for Medicaid/CHIP, it may have a spillover effect. Studies show a link between expanding parent Medicaid eligibility and growth in children’s health coverage [21]. Still, we performed a sensitivity analysis excluding patients less than 18 years of age. Next, we restricted our sample period from 2011 to the third quarter of 2015. Since the dataset shifted from reporting ICD-9 to ICD-10 after the third quarter of 2015, the classification of DKA may not be entirely consistent. In addition, the ACA was enacted in 2010 and had several other provisions that became effective long before 2014. Thus, we excluded 2010 to rule out those initial provisions that could affect diabetes outcomes. Finally, even though patients older than 65 years were excluded, we still had a sizable share of Medicare discharges. Patients younger than 65 years and eligible for Medicare are likely to have different characteristics than the rest of the sample. Thus, we repeated our analysis by excluding Medicare discharges.

We also conducted a falsification test to show whether there were significant changes in DKA incidences among patients not directly affected by Medicaid expansion. It is necessary to rule out other factors that could influence DKA trends and, thus, contaminate the results.

## Results

A total of 89,184 inpatient hospital discharge records were obtained for the city of Texarkana from the Texas DSHS Hospital Discharge Database. Table 1 reports the characteristics of the sample for the pre-expansion and post-expansion periods. Slightly less than half of the patients resided in Arkansas. In the baseline period (2010-2013), the distribution of patients into insurance categories was different. Although the share of Medicare and Uninsured was similar, patients from Arkansas were more likely to have Medicaid while those from Texas were more likely to have private insurance. The age distributions were also slightly different. The share of patients in the race category of ”White” was similar on both sides of the city. The Texas side had a higher share of patients in the ”Black” category, while the Arkansas side had a higher share of patients in the ”Other” category. The percentage of patients in different sex categories was similar. The average Charlson Comorbidity Index for patients from Texas was higher than that for patients from Arkansas. Patients from Texas were from areas with higher median incomes.

**Table 1:** Sample characteristics The distributions or means of variables by the group in the pre-expansion (2010-2013) and post-expansion (2014-2017) periods. The groups comprise patients from Arkansas (treatment) and patients from Texas (control)

	Pre-expansion (2010-2013)		Post-expansion (2014-2017)	
	Arkansas	Texas	Arkansas	Texas
Frequency	22,171	24,340	21,115	21,558
Percent	48	52	49	51
Age (%)				
5-9 years	1.39	1.06	0.64	0.69
10-14 years	0.90	0.81	0.48	0.52
15-17 years	1.87	1.77	1.15	1.38
18-19 years	3.42	3.27	2.86	3.06
20-24 years	10.75	10.33	10.56	10.75
25-29 years	9.49	9.43	9.66	9.51
30-34 years	7.11	7.54	7.57	7.68
35-39 years	6.09	5.56	5.74	5.52
40-44 years	5.90	6.13	5.22	5.84
45-49 years	7.42	7.61	7.38	6.89
50-54 years	10.19	9.65	9.69	9.25
55-59 years	12.62	13.25	12.91	12.53
60-64 years	15.27	15.26	15.77	15.98
HIV and drug/alcohol use (%)				
<17 years	0.05	0.02	0.01	0.02
18-44 years	2.72	3.23	3.84	3.80
45-64 years	4.80	5.07	6.51	6.57
Insurance type (%)				
Medicare	18.10	17.71	17.69	15.94
Medicaid	23.90	22.39	29.87	26.25
Private	47.11	48.49	48.34	45.77
Uninsured	10.90	11.41	4.09	12.03
Race (%)				
White	61.09	61.12	64.01	64.15
Black	27.31	28.90	27.77	28.00
Other	11.59	9.98	8.22	7.84
Sex (%)				
Female	67.54	68.01	67.92	67.48
Male	32.46	31.99	32.08	32.52
Charlson Comorbidity Index	0.65	0.69	0.72	0.74
Median income ($)	44,238	50,797	42,994	49,947

There were significant changes in the payer mix from the pre-expansion to post-expansion periods. The share of Medicaid discharges increased both among Texas and Arkansas patients, but the increase was bigger among Arkansas patients. The fact that the Medicaid share increased in both states implies that a woodwork effect is present. The share of privately insured increased among Arkansas patients but decreased for those from Texas. This increase is intuitive as Arkansas expanded Medicaid through the private option. Even though the share of uninsured was very similar in the baseline period, it decreased significantly among Arkansas patients but slightly increased among those from Texas following the expansion. There were no substantial demographic changes between the two time periods.

Table 2 shows average DKA incidences in the pre and post-expansion periods for both groups, as well as the changes in the averages. The average DKA incidences increased among both Arkansas (by 4.33) and Texas residents (by 8.28) from pre-expansion (2010-2013) to post-expansion period (2014-2017), which implies an upward trend in DKA hospitalizations. However, the increase was milder among the Arkansas patients with an unadjusted difference of -3.94 per 1000 discharges (95% CI: -7.28 to -0.6; p=0.021). The adjusted estimates were obtained from regressions that controlled for age category, race, sex, insurance type, the Charlson Comorbidity Index, the median income of the patient’s zip code of residence, and year-quarter dummies. The adjusted difference-in-differences of -4.17 (95% CI: -5.04 to -3.31, p<0.001) suggests that the increase in DKA hospitalizations from the pre-expansion to the post-expansion period among patients from Arkansas was significantly lower compared to those from Texas. With respect to the baseline value, it implies a 42% lower risk of hospitalization with DKA. The last column reports the results from the triple difference model. The coefficient captures if the impact of the Medicaid expansion on the outcome variable was different for patients from areas where the median household income was less than $20,864. The adjusted coefficient was -13.47 (95% CI: -22.45 to -4.49; p=0.003), confirming our hypothesis that the decrease in DKA incidence is more pronounced for patients with low incomes.

**Table 2:** Changes in DKA incidences The average DKA incidences (per 1000 discharges) among patients from Arkansas and Texas before and after the Medicaid expansion in 2014 and regression estimates along with their p-values and 95% confidence intervals are reported. The dependent variable is hospitalizations with DKA (DKA per 1000 discharges). Adjusted results are from regressions controlling for age category, race, sex, insurance type, the Charlson Comorbidity Index, the median income of the patient’s zip code of residence, and year-quarter dummies DKA: diabetic ketoacidosis; Diff: difference (post-pre); Diff-diff: difference-in-differences; Diff-diff-diff: difference-in-difference-in-differences; CI: confidence interval

	Arkansas			Texas			Diff-diff		Diff-diff-diff
	Pre	Post	Diff	Pre	Post	Diff	Unadjusted	Adjusted	Adjusted
Average/change	9.97	14.3	4.33	16.68	24.96	8.28	-3.94	-4.17	-13.47
P-value							0.021	<0.001	0.003
95% CI							(-7.28, -0.6)	(-5.04, -3.31)	(-22.45, -4.49)

Table 3 reports the sensitivity analyses, which support our earlier findings. The first column excludes patients younger than 18 years of age. The second column only covers the period from the first quarter of 2011 to the third quarter of 2015. The last column excludes patients with Medicare. All the estimated changes were statistically significant (-3.98, p<0.001; -6.02, p=007; -6.88, p=0.028), and compared to the baseline values, imply 40%, 58%, and 75% decrease, respectively. The results suggest a sizable decrease in DKA compared to what the trend would have been if Arkansas had not expanded Medicaid.

**Table 3:** Sensitivity analyses: adjusted differences in DKA incidences The adjusted difference-in-differences estimates along with their p-values and 95% CI are reported. The dependent variable is hospitalizations with DKA (DKA per 1000 discharges). All regressions were controlled for age category, race, sex, insurance type, the Charlson Comorbidity Index, the median income of the patient’s zip code of residence, and year-quarter dummies. The first column reports the results by excluding patients younger than 18. The second column reports the results for the restricted sample from the first quarter of 2011 (1Q2011) to the third quarter of 2015 (3Q2015). The last column reports the results for the sample excluding patients with Medicare. The baseline values are the average DKA incidences among patients from Arkansas before the Medicaid expansion DKA: diabetic ketoacidosis; CI: confidence interval

	(Excludes age <18 years)	(1Q2011-3Q2015)	(Excludes Medicare)
Change	-3.98	-6.02	-6.88
P-value	<0.001	0.007	0.028
95% CI	(-4.81, -3.15)	(-10.40, -1.64)	(-12.78, -0.99)
Baseline value	9.89	10.36	9.14

We also estimated the difference-in-differences model separately for each insurance type (we included patients over the age of 65 years to capture all Medicare records). This provides a falsification test as to whether there are significant changes in DKA incidences among patients not directly affected by Medicaid expansion (e.g., Medicare patients). Table 4 shows that DKA rates were lower for Medicaid and privately insured patients from Arkansas, although the latter lacked statistical power. The estimates imply that DKA incidences decreased by 36% among Medicaid patients from Arkansas compared to those from Texas (p=0.039). However, the expansion did not change DKA rates differentially among Medicare and uninsured patients from the two states. The results demonstrate that there were no other factors that influenced DKA trends. Hence, the differential changes we found are attributable to the Medicaid expansion.

**Table 4:** Adjusted differences in DKA incidences by payer type The adjusted difference-in-differences estimates along with their p-values and 95% CI are reported for each payer type as indicated in the column titles. The dependent variable is hospitalizations with DKA (DKA per 1000 discharges). All regressions are controlled for age category, race, sex, insurance type, the Charlson Comorbidity Index, the median income of the patient’s zip code of residence, and year-quarter dummies. The baseline values are the average DKA incidences among patients within corresponding insurance types from Arkansas before the Medicaid expansion DKA: diabetic ketoacidosis; CI: confidence interval

	Medicare	Medicaid	Private	Uninsured
Change	2.01	-2.92	-7.61	-1.54
P-value	0.402	0.039	0.163	0.594
95% CI	(-3.32, 7.35)	(-5.62, -0.22)	(-19.13, 3.92)	(-8.5, 5.42)
Baseline value	3.48	8.23	5.61	20.88

## Discussion

We analyzed 89,184 inpatient discharge records from hospitals in Texarkana, a two-state city that presents a natural experiment where the population on the Arkansas side gained access to expanded Medicaid benefits while those on the Texas side did not. We used difference-in-differences and triple-difference methods to examine if Medicaid expansion was associated with changes in DKA hospitalization. The first method compared the changes in DKA rates from pre-expansion to post-expansion period for the treatment (patients from Arkansas) and control (patients from Texas) groups. Even though raw DKA numbers increased over time among patients from both sides of the city, the difference-in-differences estimates indicated that DKA hospitalizations among Arkansas patients were lower by 4.17 (per 1000 discharges), adjusting for patient characteristics and year-quarter indicators. This was not an absolute decrease in the DKA cases but rather a decrease compared to the predicted trend in the counterfactual case of no Medicaid expansion. The estimated coefficient implies a 42% decrease compared to the baseline averages (p<0.001). These findings suggest that the expansion of Medicaid improved health outcomes as low-income adults who were ineligible for Medicare gained access to healthcare. People with diabetes are more likely to manage it better when they have access to healthcare (through expanded insurance coverage) and are less likely to have complications and get hospitalized with DKA. This interpretation is supported by our triple difference analysis, which examined difference-in-differences separately for people from areas with low median household income. The regression estimate (-13.47, p=0.003) is bigger in absolute value, suggesting that the decrease in DKA hospitalizations was higher among patients from low-income areas. The ACA Medicaid expansion was designed to address high uninsured rates among low-income adults who fall into the coverage gap. The findings of this study confirm and complement the earlier studies that found that the ACA Medicaid expansion was associated with improved care and control of diabetes [15-17].

Although our results have interesting policy and practical implications, the current study relied on observational administrative data with inherent limitations. For example, the providers have until the next quarter (following the discharge) to submit their data. Thus, each quarter may contain some discharges dated in the previous quarter. Also, race-related information is generally not collected by hospitals and may be subjectively captured. The data are cross-sectional since we cannot follow patients over time. Instead, we observe a sample of discharges each period. We implicitly assume that the average characteristics of patients do not change significantly between the periods.

The treatment and control groups in our analysis were dissimilar in some aspects. However, the differences-in-differences method does not require that the two groups be homogenous. The differences between treatment and control groups are acceptable as long as no other factor affects the two groups differentially before and after the treatment. The parallel trends test (available in the appendix) and the falsification test address both issues and provide evidence that the difference-in-differences methodology is a suitable one.

## Conclusions

Our results show that Medicaid expansion is associated with significantly lower rates of DKA. Also, the decline in the rate of DKA is more substantial for patients from low-income areas. This study supports the literature on the positive effects of ACA Medicaid expansion on health disparities due to socioeconomic status as it reduces the risk of DKA, a severe diabetes complication, especially among low-income patients.

## Appendices

Difference-in-differences

The patients from Arkansas are the treatment group, and those from Texas are the control group. The treatment is the Medicaid expansion implemented in Arkansas starting in the first quarter of 2014. We estimate the following statistical model:

$y_{it}=\beta_0+\beta_1{post_t}+\beta_2{treated_i}+\beta_3{post_t*treated_i}+\psi X_{it}+\gamma_t+\epsilon_{it}$

where $y_{it}$ is a dummy variable and takes the value one if the admission $i$ in period $t$ is for DKA, and zero otherwise. $post_t$ is the period after 2014 when the policy became effective. $treated_i$ is an indicator for patients from the Arkansas side of the city. The parameter of interest is $\beta_3$, which captures the differential effect of the Medicaid expansion on the treatment group. $X_{it}$​​​​​​​ is the vector of patient characteristics, including age categories, sex, race, insurance type, the Charlson Comorbidity Index, and median income at the patient’s zip code. We also include year-quarter dummies ($\gamma_t$​​​​​​​). The coefficients reported in the paper are multiplied by 1000 to reflect estimates per 1000 discharges.

Difference-in-difference-in-differences

We created an indicator variable, $low$, which takes the value of one if the median income at the patient’s zip code is less than the 10^th ^percentile of the median income distribution in the data ($20,864). We estimate the following model:

$y_{it}=\beta_0+\beta_1{post_t} +\beta_2{treated_i}+\beta_3{low_i}+\beta_4{post_t*treated_i}+\beta_5{post_t*low_i}+ \beta_6{treated_i*low_i}+\beta_7{post_t*treated_i*low_i}+\psi X_{it}+\gamma_t+\sigma_{it}$

where $y_{it}$ is a dummy variable and takes the value one if the discharge $i$ in period $t$ has DKA on record, and zero otherwise. $post_t$ is the period after 2014 when the policy became effective. $treated_i$​​​​​​​ is an indicator for patients from the Arkansas side of the city. The parameter of interest $\beta_7$ captures if the individuals from economically disadvantaged areas in the Arkansas side of the city have a different outcome after the Medicaid expansion compared to those on the Texas side. The coefficients reported in the manuscript are multiplied by 1000 to reflect estimates per 1000 discharges.

Parallel trends

The validity of difference-in-differences estimations requires the treatment and control groups to have similar trends before the treatment. We test the parallel trends assumption by estimating the following empirical equation for the pre-treatment period (Admissions not directly affected by Medicaid expansion, i.e., Medicare patients and those younger than 18 are excluded):

$y_{it}=\beta_0+\beta_1treated_i+\sum_{t=2010,1}^{2013,4}\beta_{2t}(time_t)+\sum_{t=2010,1}^{2013,4}\beta_{3t}(time_t)*treated_i+\epsilon_{it}$

where $y_{it}$ is a dummy variable and takes the value one if the discharge $i$ in period $t$ has DKA on record, and zero otherwise. $treated_i$ is an indicator for patients from the Arkansas side of the city. $time$ represents year-quarter dummies from the first quarter of 2010 (2010,1) to the fourth quarter of 2013 (2013,4). We test if $\beta_{3t}=0, \forall t$, i.e., if the coefficients of the interactions between treatment and year-quarter dummies are jointly significant. Failure to reject the null hypothesis provides evidence that our model satisfies the parallel trends assumption. The associated p-value for the F statistics is 0.3323, hence we cannot reject the null hypothesis.
